# Heart Rate Variability and Autonomic Modulations in Preeclampsia

**DOI:** 10.1371/journal.pone.0152704

**Published:** 2016-04-04

**Authors:** Shaza M. Musa, Ishag Adam, Mohamed F. Lutfi

**Affiliations:** 1 Faculty of Medicine and Health Sciences, Al Neelain University, Khartoum, Sudan; 2 Faculty of Medicine, University of Khartoum, Khartoum, Sudan; Shanghai Institute of Hypertension, CHINA

## Abstract

**Background:**

Although the exact pathophysiology of preeclampsia is not well understood, autonomic nervous system imbalance is suggested as one of the main factors.

**Aims:**

To investigate heart rate variability (HRV) and autonomic modulations in Sudanese pregnant women with preeclampsia.

**Subjects and Methods:**

A case-control study (60 women in each arm) was conducted at Omdurman Maternity Hospital—Sudan, during the period from June to August, 2014. Cases were women presented with preeclampsia and healthy pregnant women were the controls. Studied groups were matched for important determinants of HRV. Natural logarithm (Ln) of total power (TP), high frequency (HF), low frequency (LF) and very low frequency (VLF) were used to determine HRV. Normalized low and high frequencies (LF Norm and HF Norm) were used to evaluate sympathetic and parasympathetic autonomic modulations respectively.

**Results:**

Patients with preeclampsia achieved significantly higher LF Norm [49.80 (16.25) vs. 44.55 (19.15), P = 0.044] and LnLF/HF [0.04 (0.68) vs. -0.28 (0.91), P = 0.023] readings, but lower HF Norm [49.08 (15.29) vs. 55.87 (19.56), P = 0.012], compared with healthy pregnant women. Although all other HRV measurements were higher in the patients with preeclampsia compared with the controls, only LnVLF [4.50 (1.19) vs. 4.01 (1.06), P = 0.017] and LnLF [4.01 (1.58) vs. 3.49 (1.23), P = 0.040] reached statistical significance.

**Conclusion:**

The study adds further evidence for the dominant cardiac sympathetic modulations on patients with preeclampsia, probably secondary to parasympathetic withdrawal in this group. However, the higher LnVLF and LnLF readings achieved by preeclamptic women compared with the controls are unexpected in the view that augmented sympathetic modulations usually depresses all HRV parameters including these two measures.

## Introduction

Preeclampsia is a multi-systemic disorder characterized by hypertension, proteinuria and/or end-organ dysfunction after 20 weeks of gestation [1]. Manifestations of preeclampsia are mostly attributed to placental ischemia that results in release of certain antiangiogenic substances into the circulation and ultimately endothelial dysfunction [2]. Although hypertension associated with preeclampsia can be explained by endothelial dysfunction [3], its relation to the concomitant derangement of neural control of cardiovascular system remains to be explored by further investigations [4]. Several studies were conducted to evaluate pattern of autonomic activity in pregnant and preeclamptic women based on levels of catecholamines [5–8]. Although some researchers were able to demonstrate higher resting plasma catecholamines levels and consequently increased sympathetic activity in preeclampsia compared with normal pregnancy [5, 6], other studies either failed to reproduce such differences [7] or prove the reverse [8]. Recent reports on autonomic balance are mostly based on evaluation of the modulatory effects of sympathetic and parasympathetic nervous systems on the heart rate (HR) [9–11] and the blood pressure [12].

Heart rate variability (HRV) is considered one of the most informative tools for assessment of cardiac autonomic modulations [13]. HRV is frequently used as a prognostic measurement for cardiac diseases [11, 13] and it is currently used to evaluate the impact of autonomic imbalance on certain diseases [14–17].

Most studies based on these new techniques, namely HRV and blood pressure variability (BPV), suggest that pregnancy per se shifts cardiac autonomic balance towards sympathetic dominance [9, 10] and this shift is even more prominent if pregnancy is complicated with preeclampsia [18]. In contrast, other studies failed to distinguish autonomic modulations of normal pregnancy from pregnancies complicated by preeclampsia based on HRV derived values [19]. The previous studies evaluating HRV in preeclampsia had not considered variations in HR as a possible confounder while comparing HRV parameters between the studied groups, which may explain their contradictory findings [18, 19]. Furthermore, confounders like age [20, 21], body mass index (BMI) [22], hemoglobin concentration [23], gestational age [24] and HR [13] that might have possible influences on HRV were not considered when comparing cardiac autonomic modulations between the studied groups.

There are few studies exploring pattern of HRV in preeclampsia and none of them was conducted in Sudan, where preeclampsia/eclampsia is the main cause of maternal mortality [25]. The aim of the present study is to investigate HRV and autonomic modulations in pregnant Sudanese women with preeclampsia and to add to the recent studies on pathophysiology of preeclampsia [26–29].

## Materials and Methods

A case-control study was conducted at Omdurman Maternity Hospital—Sudan, during the period from June to August, 2014. Cases were women presented with preeclampsia, which is defined as the occurrence of hypertension (systolic blood pressure ≥140 mm Hg or diastolic blood pressure ≥ 90 mm Hg) after 20 weeks of gestation in woman who is normotensive before, and proteinuria (presence of 300 mg or more of protein in 24 h urine sample or ≥ 2+ on dipstick) [1]. Preeclampsia was considered mild or severe according to the diastolic blood pressure of < 110, or ≥110 mmHg respectively. The controls were healthy pregnant women. Women with thyroid disease, hypertension, renal disease, diabetes, liver disease and those who received medication for hypertension were excluded.

After signing an informed consent, medical and obstetrics history (age, parity, and gestational age) was gathered and recorded. Weight and height were measured and BMI was calculated via weight in kilograms divided by the square of height in meters. Systolic and diastolic blood pressures were assessed in supine position using mercury sphygmomanometer by the same investigator (SMM). All women were enrolled during the morning hours (8 AM -12 PM) and before receiving any medications.

HRV parameters were derived from 5-min electrocardiogram (ECG) recordings in the supine position using clean ECG signals during comfortable breathing without movement artifacts. The Biocom 3000 ECG recorder also calculated the mean heart rate (MHR) at the time of ECG recording as recently described [17]. Time and frequency domains analysis were used to determine HRV and cardiac autonomic modulations in both cases and controls. Natural logarithm (Ln) of the standard deviation of the NN intervals (LnSDNN), the square root of the mean squared differences of successive NN intervals (LnRMSSD), total power (LnTP), very low frequency (LnVLF), low frequency (LnLF) and high frequency (LnHF) were used to evaluate HRV. Normalized low frequency (LF Norm) and high frequency (HF Norm) were used to determine sympathetic and parasympathetic autonomic modulations respectively [11, 13, 17]. LnTP and LnSDNN broadly reflect the overall HRV [13]. LnRMSSD correlate well with LnHF and both are commonly used to assess the influence of parasympathetic regulation of the heart [11]. The physiological basis of the LnVLF is controversial, but is likely influenced by parasympathetic outflow [11, 13]. LnLF is usually used as an indicator of sympathetic modulations, although some reports claimed it is a parasympathetic index [11, 13].

A sample size of 60 women in each arm of the study was calculated to give the significant difference in the mean of the HRV parameters with 80% power and a difference of 5% at α = 0.05.

The study received ethical clearance from the Research Committee of the Research Board of the Faculty of Medicine, Al Neelain University, Sudan.

Data were entered in the computer using SPSS for windows version16.0 (SPSS Inc., Chicago, IL, USA). Continuous and categorical data were compared between the two groups using t- and Chi-square tests, respectively. Using a general linear model, MHR was introduced as a covariate while comparing HRV parameter between the studied groups. Linear regression analyses were conducted where (Ln) of HRV parameters were the dependent variables and socio-demographic, clinical and biochemical characteristics were the independent variables. P < 0.05 was considered significant.

## Results

The two groups were well matched in their age, gestational age, body mass index, hemoglobin concentration and MHR, Table 1.

**Table 1:** Comparison between the mean (SD) of the studied variables between preeclamptic and control women.

Variables	Preeclampsia (n = 60)	Controls (n = 60)	P
Age, years	30.6 (6.0)	30.0(6.2)	0.582
Gestational age, weeks	33.8(4.3)	32.9(4.0)	0.213
Body mass index, kg/m^2^	31.0(6.3)	29.7(6.1)	0.256
Hemoglobin, g/dl	11.6(1.5)	11.7(1.0)	0.735
Systolic blood pressure, mm/Hg	172.5(16.8)	116.6(9.3)	< 0.001
Diastolic blood pressure, mm/Hg	108.1(9.8)	74.7(6.7)	< 0.001
MHR, beat /minute	95.1(15.8)	96.0(12.7)	0.721

There was no significant difference in LnSDNN, LnRMSSD, LnTP, and LnHF between patients with preeclampsia and the controls, Table 2.

**Table 2:** Comparison of mean (SD) of HRV measurements between preeclamptic and control women.

	Preeclampsia	Controls	P	
Variables	N = 60	N = 60	Not Adjusted for MHR	Adjusted for MHR
LnSDNN	3.74 (0.80)	3.50 (0.55)	0.056	0.053
LnRMSSD	3.76 (0.75)	3.60 (0.59)	0.198	0.214
LnTP (ms^2^/Hz)	5.40 (1.45)	4.99 (1.10)	0.088	0.077
LnVLF (ms^2^/Hz)	4.50 (1.19)	4.01 (1.06)	0.019	0.017
LnLF (ms^2^/Hz)	4.01 (1.58)	3.49 (1.23)	0.047	0.040
LnHF (ms^2^/Hz)	3.93 (1.87)	3.71 (1.34)	0.463	0.523
LF Norm (nu)	49.80 (16.25)	44.55 (19.15)	0.108	0.044
HF Norm (nu)	49.08 (15.29)	55.87 (19.56)	0.036	0.012
LnLF/HF	0.04 (0.68)	-0.28 (0.91)	0.033	0.023

**Fig 1:**
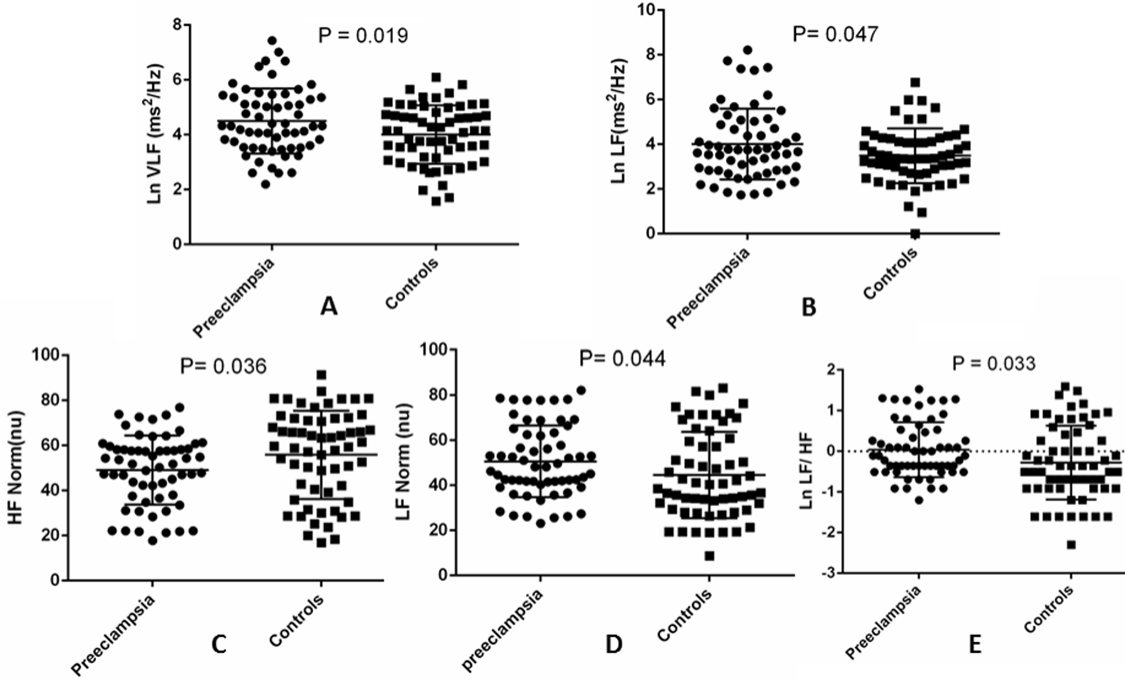
Comparison of (A) LnVLF, (B) LnLF, (C) LF Norm, (D) HF Norm and (E) LnLF/HF between preeclamptic and control women.

In comparison with the controls, patients with preeclampsia had significantly higher LnVLF [4.50 (1.19) vs. 4.01 (1.06), ms^2^/Hz, P = 0.019], LnLF [4.01 (1.58) vs. 3.49 (1.23) ms^2^/Hz, P = 0.047], LF Norm [49.80 (16.25) vs. 44.55 (19.15) nu, P = 0.044] and LnLF/HF [0.04 (0.68) vs. -0.28 (0.91), P = 0.033], Table 2, Fig 1. In contrast, HF Norm was significantly lower in patients with preeclampsia [49.08 (15.29) nu] compared with the controls [55.87 (19.56) nu, P = 0.023], Table 2, Fig 1.

All HRV measurements were not different when patients with mild preeclampsia were compared with those with severe form of the disease, Table 3.

**Table 3:** Comparison of mean (SD) of HRV measurements between women with severe and mild preeclampsia.

	Severe preeclampsia	Mild preeclampsia	P	
Variables	N = 60	N = 60	Not Adjusted for MHR	Adjusted for MHR
LnSDNN	3.7(0.7)	3.8(0.8)	0.716	0.995
LnRMSSD	3.8(0.8)	3.5(0.5)	0.221	0.085
LnTP (ms^2^/Hz)	5.4(1.5)	5.2(0.9)	0.557	0.244
LnVLF (ms^2^/Hz)	4.4(1.2)	4.6(0.8)	0.548	0.728
LnLF (ms^2^/Hz)	4.0(1.7)	3.8 (0.9)	0.566	0.245
LnHF (ms^2^/Hz)	4.0(2.0)	3.6(1.3)	0.549	0.206
LF Norm (nu)	4.0(1.7)	3.8(0.9)	0.566	0.341
HF Norm (nu)	4.0(2.0)	3.6(1.3)	0.549	0.347
LnLF/HF	0.008(0.6)	0.1(0.7)	0.560	0.363

In linear regression, preeclampsia was significantly associated with LnVLF (0.461 ms^2^/Hz, P = 0.023) and LnLF (─ 0.681 ms^2^/Hz, P = 0.013) after adjusted for MHR, Table 4.

**Table 4:** Factors associated with LnVLF and LnLF (ms^2^/Hz) in preeclamptic and controls pregnant women using linear regression analyses.

Variable	LnVLF (ms^2^/Hz)			LnLF (ms^2^/Hz)		
	Coefficient	SE	P-value	Coefficient	SE	P-value
Age, year	─ 0.018	0.017	0.289	─ 0.041	0.276	0.072
Parity	0.118	0.241	0.625	─ 0.601	3.961	0.071
Gestational age, weeks	─ 0.006	0.027	0.832	0.017	0.041	0.646
Body mass index, (kg)/ (m)^2^	─ 0.010	0.016	0.555	─ 0.011	0.265	0.612
Hemoglobin, g/dl	0.051	0.071	0.479	0.040	1.169	0.680
Preeclampsia*	0.461	0.199	0.023	─ 0.681	3.269	0.013
Mean heart rate	─ 0.029	0.006	<0.001	─ 0.047	0.008	0.013

SE = standard error

*adjusted for MHR

Likewise, preeclampsia was significantly associated with HF Norm (7.143, nu, P = 0.039) and LnLF/HF (─ 0.311, P = 0.038), Table 5.

**Table 5:** Factors associated with LF Norm, HF Norm and Ln LF/ HF in preeclamptic and controls pregnant women using linear regression analyses.

Variable	LF Norm (nu)			HF Norm (nu)			Ln LF/ HF		
	Coefficient	SE	P-value	Coefficient	SE	P-value	Coefficient	SE	P-value
Age, year	─ 0.338	0.271	0.215	0.360	.286	0.212	─ 0.014	0.012	0.255
Parity	─ 2.837	3.951	0.474	2.998	4.162	0.473	─ 0.197	0.181	0.278
Gestational age, weeks	1.011	0.436	0.022	─ 0.985	0.459	0.034	0.046	0.020	0.024
Body mass index,(kg/m^2^)	0.284	0.265	0.287	─ 0.332	0.280	0.238	0.015	0.012	0.224
Hemoglobin, g/dl	1.138	1.161	0.329	─ 0.277	1.221	0.821	0.037	0.053	0.486
Preeclampsia*	─ 5.707	3.239	0.081	7.143	3.418	0.039	─ 0.311	0.148	0.038
Mean heart rate	0.351	0.109	0.002	─ 0.311	0.112	0.006	0.015	0.005	0.003

SE = standard error

*adjusted for MHR

## Discussion

The main finding of the current study was the different patterns of HRV and autonomic modulations in patients with preeclampsia compared with healthy pregnant women. The higher LF Norm and LnLF/HF readings in preeclamptic women compared with the controls point to augmented cardiac sympathetic modulations in the first group. Enhanced sympathetic modulation in patients with preeclampsia is likely due to parasympathetic withdrawal among patients with preeclampsia as indicated by the lower HF Norm values they achieved. The trend of cardiac autonomic modulations of patients with preeclampsia is comparable with the recent studies in the field [12, 30], but not others [19]. Noteworthy, the interpretation of cardiac autonomic modulations of the present results was largely based on normalized LF and HF which, in contrast to absolute values of LF and HF powers, are inversely proportional. Normalized LF and HF are therefore considered as better indicators of sympathetic and parasympathetic modulations respectively [17, 19].

Yang et al observed higher LF/HF and LF, but lower HF, in pregnant compared with the non-pregnant women [18]. In addition, the preeclamptic group demonstrated lower HF, but higher LF/HF, compared with the non-pregnant as well as normal pregnant women [18]. The study concluded that normal pregnancy readjust autonomic modulation towards predominance of sympathetic over parasympathetic tone and this readjustment is further augmented if the pregnant women developed preeclampsia [18]. In a recent report, the enhanced cardiac sympathetic tone persists following delivery in cases with past history of preeclampsia, but not normal pregnancy [30]. The attenuated parasympathetic modulations of patients with preeclampsia showed in our results and Yang et al study were also approved in a naive research conducted on 385 American military women to determine the relationship between HRV and the incidence of hypertensive diseases of pregnancy [12]. In contrast, an old study designed by Eneroth and Storck failed to demonstrate differences in HRV between normal pregnant women and patients with preeclampsia [19]. According to Eneroth and Storck results, pregnant women with preeclampsia had prolonged NN intervals during daytime compared to the controls; however, all frequency domain HRV measures were comparable in the studied groups.

In the current study; all absolute values of time and frequency domains measures are higher in the patients with preeclampsia compared with the control group; however, only LnVLF and LnLF achieve statistical significance. Higher LnVLF and LnLF readings attained by preeclamptic women are interesting because attenuated parasympathetic modulations in this group are expected to depress all HRV parameters including these two measures [31, 32]. In an experiment exploring the mechanism of VLF oscillations, parasympathetic blockade decreased VLF band of frequency domain HRV by 92% [33]. The absolute LF power was accepted by many authorities as an indicator of sympathetic modulations [34–36]. However, recent studies demonstrated that the absolute value of LF is mainly determined by baroreflex and consequently reflect parasympathetic influences on the heart [31, 36]. Based on the foregoing narrative, the significantly high LnVLF and LnLF in patients with preeclampsia compared with the normal pregnant women in spite of lower parasympathetic modulations in first group is mysterious and should motivate researchers for further investigations.

Previous studies showed important effects of age [20, 21], BMI [22], hemoglobin concentration [23], gestational age [24] and MHR [11, 13] on HRV. In the present study, gestational age and MHR, but not the other confounders we matched for, significantly affect the variations in indicators of autonomic modulations observed among the studied groups. According to our result, HF Norm decreases, but LF Norm and LnLF/HF increases, with gestational age. Comparable pattern of change in HRV with gestational age was observed by Tejera et al while evaluating HRV among normal, hypertensive and preeclamptic pregnant women [24]. According to Tejera et al, HF significantly decreases while all other frequency domain parameters linearly increase with gestational age. Other studies were able to demonstrate significant rise of LF in the third trimester compared with earlier stages of pregnancy in women who later developed preeclampsia [37, 38]. Alternatively, the influence of MHR on HRV is not only physiological, but also mathematical [39]. Due to the non-linear relationship between MHR and RR interval, HRV is enhanced by lower values of MHR and vice versa [40]. This fact explains the extremely significant association between MHR and HRV parameters subjected to regression analysis in our study. It also explains why MHR was introduced as a covariate while comparing HRV of different groups in the present study and others [15, 17].

In the current study women were enrolled before starting any anti-hypertensive medications. Previous studies showed either negative influence [24] or no effect [41] of antihypertensive treatments of preeclampsia on LF band of frequency domain HRV and consequently sympathetic tone.

A potential limitation of the present study is that it did not consider other measurements of autonomic activity. If done, concurrent evaluation of sympathetic/parasympathetic activity by other techniques could have offered other tools for further strengthening of the present conclusions.

## Conclusion

This study adds further evidence for the dominant cardiac sympathetic modulations in patients with preeclampsia compared with normal pregnant women, probably due to parasympathetic withdrawal in the first group. However, the higher LnVLF and LnLF readings achieved by preeclampsia pregnant women compared with the controls are unexpected in the view that augmented sympathetic modulations usually depresses all HRV parameters including these two measures. Explanation of this contradiction remains to be uncovered by further researches.

## References

[1] American College of Obstetricians and Gynecologists; Task Force on Hypertension in Pregnancy. Hypertension in pregnancy. Report of the American College of Obstetricians and Gynecologists’ Task Force on Hypertension in Pregnancy. Obstet Gynecol. 2013; 122(5):1122–31. 10.1097/01.AOG.0000437382.03963.88 24150027

[2] Maynard SE, Karumanchi SA. Angiogenic factors and preeclampsia. Semin Nephrol 2011; 31(1):33–46. 10.1016/j.semnephrol.2010.10.004 21266263 PMC3063446

[3] Lamarca B. Endothelial dysfunction. An important mediator in the pathophysiology of hypertension during pre-eclampsia. Minerva Ginecol. 2012; 64(4):309–20. 22728575 PMC3796355

[4] Greenwood JP, Scott EM, Stoker JB, Walker JJ, Mary DA. Sympathetic neural mechanisms in normal and hypertensive pregnancy in humans. Circulation. 2001; 104(18):2200–4. 11684631 10.1161/hc4301.098253

[5] Natrajan PG, McGarrigle HH, Lawrence DM, Lachelin GC. Plasma noradrenaline and adrenaline levels in normal pregnancy and in pregnancy-induced hypertension. Br J Obstet Gynaecol. 1982; 89(12):1041–5. 7171514 10.1111/j.1471-0528.1982.tb04661.x

[6] Davey DA, Macnab MF. Plasma adrenaline, noradrenaline and dopamine in pregnancy hypertension. Br J Obstet Gynaecol. 1981; 88(6):611–8. 7248219 10.1111/j.1471-0528.1981.tb01217.x

[7] Pedersen EB, Rasmussen AB, Christensen NJ, Johannesen P, Lauritsen JG, Kristensen S, et al Plasma noradrenaline and adrenaline in pre-eclampsia, essential hypertension in pregnancy and normotensive pregnant control subjects. Acta Endocrinol (Copenh). 1982; 99(4):594–600. 7072455 10.1530/acta.0.0990594

[8] Tunbridge RD, Donnai P. Plasma noradrenaline in normal pregnancy and in hypertension of late pregnancy. Br J Obstet Gynaecol. 1981; 88(2):105–8. 7459298 10.1111/j.1471-0528.1981.tb00950.x

[9] D'Silva LA, Davies RE, Emery SJ, Lewis MJ. Influence of somatic state on cardiovascular measurements in pregnancy. Physiol Meas. 2014; 35(1):15–29. 10.1088/0967-3334/35/1/15 24345774

[10] Matsuo H, Inoue K, Hapsari ED, Kitano K, Shiotani H. Change of autonomic nervous activity during pregnancy and its modulation of labor assessed by spectral heart rate variability analysis. Clin Exp Obstet Gynecol. 2007; 34(2):73–9. 17629156

[11] Task Force of the European Society of Cardiology and the North American Society of Pacing and Electrophysiology Heart rate variability standards of measurement, physiological interpretation and clinical use. Circulation. 1996; 93:65–1043. 8598068

[12] Flood P, McKinley P, Monk C, Muntner P, Colantonio LD, Goetzl L, et al Beat-to-beat heart rate and blood pressure variability and hypertensive disease in pregnancy. Am J Perinatol. 2015; 32(11):1050–8. 10.1055/s-0035-1548542 25970272

[13] Lutfi MF. Review article: Heart rate variability. Sud JMS. 2011; 6:43–50.

[14] Alonso A, Huang X, Mosley TH, Heiss G, Chen H. Heart rate variability and the risk of Parkinson disease: The Atherosclerosis Risk in Communities study. Ann Neurol. 2015; 77(5):877–83. 10.1002/ana.24393 25707861 PMC4529999

[15] Lutfi MF. Autonomic modulations in patients with bronchial asthma based on short-term heart rate variability. Lung India. 2012; 29(3):254–8. 10.4103/0970-2113.99111 22919165 PMC3424865

[16] Engel T, Ben-Horin S, Beer-Gabel M. Autonomic Dysfunction Correlates with Clinical and Inflammatory Activity in Patients with Crohn's Disease. Inflamm Bowel Dis. 2015; 21(10):2320–6. 10.1097/MIB.0000000000000508 26181429

[17] Lutfi MF. Patterns of heart rate variability and cardiac autonomic modulations in controlled and uncontrolled asthmatic patients. BMC Pulmonary Medicine. 2015; 15:119 10.1186/s12890-015-0118-8 26459382 PMC4603942

[18] Yang CC, Chao TC, Kuo TB, Yin CS, Chen HI. Preeclamptic pregnancy is associated with increased sympathetic and decreased parasympathetic control of HR. Am J Physiol Heart Circ Physiol. 2000; 278(4):H1269–73. 10749724 10.1152/ajpheart.2000.278.4.H1269

[19] Eneroth E, Storck N. Preeclampsia and maternal heart rate variability. Gynecol Obstet Invest. 1998; 45(3):170–3. 9565140 10.1159/000009949

[20] Zhang J. Effect of age and sex on heart rate variability in healthy subjects. J Manipulative Physiol Ther. 2007; 30(5):374–9. 17574955 10.1016/j.jmpt.2007.04.001

[21] Lutfi M F, Sukkar M Y. The Effect of Gender on Heart Rate Variability. Int J Health Sciences. 2011; 5(2); 146–154. PMC3521833 23267292

[22] Lutfi MF, Sukkar MY. Relationship of height, weight and body mass index to heart rate variability. Sudan Med J. 2011; 47(1):14–19.

[23] Lutfi M F. Effect of Hemoglobin concentration on heart rate variability. Int J Pharm Bio Res. 2011; 2(5):127–131.

[24] Tejera E, Areias MJ, Rodrigues AI, Nieto-Villar JM, Rebelo I. Blood pressure and heart rate variability complexity analysis in pregnant women with hypertension. Hypertens Pregnancy. 2012; 31(1):91–106. 10.3109/10641955.2010.544801 21599453

[25] Elhassan EM, Mirghani OA, Adam I. High maternal mortality and stillbirth in the Wad Medani Hospital, Central Sudan, 2003–2007. Trop Doct. 2009; 39(4):238–9. 10.1258/td.2009.090005 19762581

[26] Elhaj ET, Adam I, Alim A, Elhassan EM, Lutfi MF. Thyroid Function/Antibodies in Sudanese Patients with Preeclampsia. Front Endocrinol (Lausanne). 2015; 6:87. 26124747 10.3389/fendo.2015.00087 PMC4464070

[27] Bueno AA, Ghebremeskel K, Bakheit KH, Elbashir MI, Adam I. Dimethyl acetals, an indirect marker of the endogenous antioxidant plasmalogen level, are reduced in blood lipids of Sudanese pre-eclamptic subjects whose background diet is high in carbohydrate. J Obstet Gynaecol. 2012;32(3):241–6 10.3109/01443615.2011.641622 22369396

[28] Bakheit KH, Bayoumi NK, Eltom AM, Elbashir MI, Adam I. Cytokines profiles in Sudanese women with preeclampsia. Hypertens Pregnancy. 2009; 28(2):224–9. 10.1080/10641950802601245 19437232

[29] Abdullahi H, Osman A, Rayis DA, Gasim GI, Imam AM, Adam I. Red blood cell distribution width is not correlated with preeclampsia among pregnant Sudanese women. Diagn Pathol. 2014; 9:29 10.1186/1746-1596-9-29 24499498 PMC3916796

[30] Murphy MS, Seaborn GE, Redfearn DP, Smith GN. Reduced Heart Rate Variability and Altered Cardiac Conduction after Pre-Eclampsia. PLoS One. 2015; 10(9):e0138664 10.1371/journal.pone.0138664 26407294 PMC4583376

[31] Hadase M, Azuma A, Zen K, Asada S, Kawasaki T, Kamitani T, et al Very low frequency power of heart rate variability is a powerful predictor of clinical prognosis in patients with congestive heart failure. Circ J. 2004; 68(4):343–7. 15056832 10.1253/circj.68.343

[32] Goldstein DS, Bentho O, Park MY, Sharabi Y. Low-frequency power of heart rate variability is not a measure of cardiac sympathetic tone but may be a measure of modulation of cardiac autonomic outflows by baroreflexes. Exp Physiol. 2011; 96(12):1255–61. 10.1113/expphysiol.2010.056259 21890520 PMC3224799

[33] Taylor JA, Carr DL, Myers CW, Eckberg DL. Mechanisms underlying very-low-frequency RR-interval oscillations in humans. Circulation. 1998; 98(6):547–55. 9714112 10.1161/01.cir.98.6.547

[34] Zhong X, Hilton HJ, Gates GJ, Jelic S, Stern Y, Bartels MN, et al Increased sympathetic and decreased parasympathetic cardiovascular modulation in normal humans with acute sleep deprivation. J Appl Physiol (1985). 2005; 98(6):2024–32. 15718408 10.1152/japplphysiol.00620.2004

[35] Perini R, Veicsteinas A. Heart rate variability and autonomic activity at rest and during exercise in various physiological conditions. Eur J Appl Physiol. 2003; 90(3–4):317–25. 13680241 10.1007/s00421-003-0953-9

[36] Lotufo PA, Valiengo L, Benseñor IM, Brunoni AR. A systematic review and meta-analysis of heart rate variability in epilepsy and antiepileptic drug. Epilepsia. 2012; 53(2):272–82. 10.1111/j.1528-1167.2011.03361.x 22221253

[37] Rang S, Wolf H, van Montfrans GA, Karemaker JM. Serial assessment of cardiovascular control shows early signs of developing pre-eclampsia. J Hypertens. 2004 2;22(2):369–76. 15076196 10.1097/00004872-200402000-00022

[38] Lewinsky RM, Riskin-Mashiah S. Autonomic imbalance in preeclampsia: evidence for increased sympathetic tone in response to the supine-pressor test. Obstet Gynecol. 1998; 91(6):935–9. 9610999 10.1016/s0029-7844(98)00105-7

[39] Sacha J, Barabach S, Statkiewicz-Barabach G, Sacha K, Müller A, Piskorski J, et al How to strengthen or weaken the HRV dependence on heart rate—description of the method and its perspectives. Int J Cardiol. 2013; 168(2):1660–3. 10.1016/j.ijcard.2013.03.038 23578892

[40] Sacha J, Pluta W. Alterations of an average heart rate change heart rate variability due to mathematical reasons. Int J Cardiol 2008; 128:444–7. 17689709 10.1016/j.ijcard.2007.06.047

[41] Lakhno IV. Antihypertensive drugs impact on the regulation of maternal and fetal cardiac activity in pregnant women with preeclampsia. The New Armenian Medical Journal. 2015; 9(1):58–62.

